# SARS-CoV-2 and Animals: From a Mirror Image to a Storm Warning

**DOI:** 10.3390/pathogens11121519

**Published:** 2022-12-12

**Authors:** Francesco Pellegrini, Ahmed Hassan Omar, Canio Buonavoglia, Annamaria Pratelli

**Affiliations:** Department of Veterinary Medicine, University of Bari, Sp Casamassima Km 3, 70010 Valenzano, BA, Italy

Coronavirus disease 2019 (COVID-19), caused by severe acute respiratory syndrome coronavirus-2 (SARS-CoV-2), emerged in Wuhan city (Hubei province, China) in December 2019, and the World Health Organization (WHO) declared an international public health emergency on 11 March 2020 [1]. Epidemiological studies have traced the origin of the virus to the Huanan Seafood Market in Wuhan where live animals are sold, leading to domestic and wild animal interactions with humans. Sequence and phylogenetic analyses have revealed a clear similarity of SARS-CoV-2 with coronavirus (CoV) in horseshoe bats (*Rhinolophus affinis*), raising questions about the animal origin of the virus and strongly suggesting that bats are likely to be involved in the emergence of SARS-CoV-2 from an ancestor CoV [2]. The *Coronaviridae* family is large and is comprised of four genera of complex RNA viruses: alphacoronavirus (αCoV), betacoronavirus (βCoV), gammacoronavirus (γCoV), and deltacoronavirus (δCoV); these are characterized by variable tissue tropism and by the ability to easily cross interspecies barriers, causing diseases with remarkable differences [3]. While αCoV and βCoV infect mammals, γCoV and δCoV primarily infect birds, with some mammalian spillover. Human CoVs (HCoVs) are often of animal origin but adapt to humans via direct jumping or through intermediate species. These data and the zoonotic origin of SARS-CoV-2 quickly raised questions concerning the potential role that animals could play in transmitting the virus to humans or other species [4].

In fact, soon after its appearance, SARS-CoV-2 was isolated from several animal hosts in many countries, and concerns about the possibility of virus transmission from humans to animals, and vice versa, rapidly emerged, undermining the changes in counteracting the spread of the pandemic [5]. To date, SARS-CoV-2 has been detected in almost 20 different animal species belonging to ten families (*Felidae*, *Viverridae*, *Hyaenidae*, *Canidae*, *Mustelidae*, *Procyonidae*, *Cervidae*, *Hippopotamidae*, *Hominidae*, and *Cricetidae*) highlighting the role of animals as natural reservoirs/intermediate hosts for new adaptable viruses with increased virulence [6,7,8].

Farm animals can be infected by SARS-CoV-2, although experimental infections in calves, pigs, and chickens have proven that they are not very susceptible [9,10,11]. On the contrary, American minks are sensitive to SARS-CoV-2, and in Denmark, all existing minks were culled, and mink farming was banned after SARS-CoV-2 infection was confirmed in twelve humans [12]. Similarly in the Netherlands, after the infection passed from minks to humans, their role as a potential intermediate host of the virus was suggested [13]. Interestingly, the SARS-CoV-2 mutation Y453F, which is responsible for immune evasion in humans, was detected in viruses isolated from minks, leading to the assumption that humans acquired the mutant directly from minks [14].

SARS-CoV-2 infection in wildlife is not frequently observed, but it has been confirmed by experimental infections in deer, raccoons, African green monkeys, rhesus monkeys, and tree shrews [11]. Ferrets are susceptible to natural and experimental infection with SARS-CoV-2; therefore, it has been hypothesized that this animal-adapted virus can be transmitted back to humans, ensuring the continuation of the disease [15]. Therefore, wild animals should be constantly and continuously monitored since surveillance and quarantine cannot be provided to prevent risk to grazing animals. Similarly, birds, despite mainly carrying γCoV and δCoV, represent possible transmitters of CoV zoonotic disease, especially given their ability to travel long distances; however, to date, no SARS-CoV-2 infection of in birds has been reported [16].

Interestingly, SARS-CoV-2 infections were reported worldwide in pets housed with COVID-19-infected humans and with confirmation that sequences obtained from pets and humans were sometimes identical, the possibility of reverse zoonosis in the case of pets cannot be ignored [17,18]. Seroepidemiological data in Japan, Hong Kong, and the USA suggest that SARS-CoV-2 infection in pets is not unusual, and health authorities have established a surveillance campaign for domestic animals in contact with human cases of COVID-19. Overall, pets affected by COVID-19 were asymptomatic or suffered from mild respiratory symptoms [2]. Cats are more susceptible than dogs and can transmit the disease to naive cats. Additionally, human–cat transmission has been documented, and a relationship was confirmed between the whole genome sequences of the viruses from the infected cat and its owner [19]. Furthermore, sequence analysis of the ACE-2 proteins in cats, golden hamsters, and rabbits has shown that only 3 of the 22 amino acid residues responsible for binding with SARS-CoV-2 are different, while in dogs, they differ in 5 residues [11,20]. Close attention should also be paid to domestic mustelids, rabbits, rodents, and lagomorphs [6].

Interestingly, in 2017–2018, a CCoV strain (HuPn-2018) closely related to canine coronavirus (CCoV) was identified in children with pneumonia in Malaysia. The virus showed a very unique deletion in the nucleoprotein, as observed in SARS-CoV and SARS-CoV-2, suggesting a zoonotic origin; this led researchers to focus on animal CCoVs, hypothesizing what could be learned from these viruses [21]. The newly identified CCoV-Hupn-2018 is another warning sign, for which research should focus on the mechanisms of recombination among CoVs, in addition to the variants that arise as mutation events. The recombination observed over the years among CCoVs are examples of the evolutionary mechanisms of these viruses and continuous, and constant monitoring of these viruses is required [7,8,22].

Despite the multitude of investigations conducted globally and on an ever-growing variety of animal species, the reservoir host of SARS-CoV-2 is still unknown. However, the current model of COVID-19 emergence (not confirmed) suggests cross-species spillover that underwent genetic refinement in an intermediate host with an ACE-2 receptor very similar to that of humans [11]. As SARS-CoV-2 infection has been reported in many animal species thus far, there is a compelling need to implement the One Health approach to establish combined surveillance programs to curb the disease.

It is therefore necessary to implement cooperation between human and animal health authorities for: (i) preventing viral transmission from humans to susceptible animals; (ii) monitoring susceptible animals; (iii) reporting cases of animal COVID-19; and (iv) sharing the genetic sequences of viruses isolated from infected animals with the global health community (https://wahis.woah.org, accessed on 8 December 2022)
